# Rapid Emergence of Oseltamivir Resistance

**DOI:** 10.3201/eid1604.091706

**Published:** 2010-04

**Authors:** Cheng Len Sy, Susan Shin-Jung Lee, Ming-Tsan Liu, Hung-Ching Tsai, Yao-Shen Chen

**Affiliations:** Kaohsiung Veterans General Hospital, Kaohsiung, Taiwan (C.L. Sy, S.S.-J. Lee, H.-C. Tsai, Y.-S. Chen); National Yang-Ming University, Taipei, Taiwan (S.S.-J. Lee, H.-C. Tsai, Y.-S. Chen); Centers for Disease Control, Taipei (M.-T. Liu)

**Keywords:** Influenza, oseltamivir, resistance, H1N1, viruses, expedited, letter, *Suggested citation for this article*: Sy CL, Lee S S-J, Liu M-T, Tsai H-C, Chen Y-S. Rapid emergence of oseltamivir resistance [letter]. Emerg Infect Dis [serial on the Internet]. 2010 Apr [*date cited*]. http://dx.doi.org/10.3201/eid1604.091706

**To the Editor:** The influenza A pandemic (H1N1) 2009 virus has spread globally since it first appeared in Mexico in April 2009. This third influenza pandemic since the Spanish influenza pandemic of 1918 (*1*) has caused at least 400,000 infections within 6 months; estimated mortality rate is 1.2% (*2*). Emergence of oseltamivir resistance in the pandemic (H1N1) 2009 virus is a rising challenge to global control of the pandemic. So far, 39 oseltamivir-resistant pandemic (H1N1) 2009 viruses have been reported worldwide (*3*). Among the 32 resistant strains reported in October 2009, a total of 13 (41%) were associated with postexposure chemoprophylaxis and 16 (50%) were from samples of patients receiving oseltamivir (*3*). We report rapid emergence of resistance (H275Y mutation) in a patient, 4 days after early treatment with standard doses of oseltamivir for pandemic (H1N1) 2009 pneumonia.

On September 1, 2009, a 20-year-old man with mental retardation consulted the emergency department of Kaohsiung Veterans General Hospital after 1 day of fever, sore throat, and nonproductive cough. A rapid diagnostic antigen test (Quick Vue Influenza test; Quidel, San Diego, CA, USA) showed the man to be positive for influenza A. He was hospitalized for bilateral pneumonitis and treated with oseltamivir (75 mg 2×/day for 5 days), ampicillin/sulbactam, and erythromycin. However, a progressive increase in bilateral perihilar interstitial infiltration developed on the third day, accompanied by increasing dyspnea. Influenza A pandemic (H1N1) 2009 virus was isolated from the patient’s nasopharyngeal secretions on days 1 and 4 by using MDCK cells. After DNA sequence analysis of the neuraminidase gene, the mutation of H275Y was not found in the first isolate, but sequence analysis of the second isolate detected mixed populations (C/T) in the 823-nt position of the neuraminidase gene. Only a single pattern (T) was found from the cultured viruses, indicating a mixed quasispecies of oseltamivir-resistant and -susceptible viruses emerging after 4 days of oseltamivir treatment. The oseltamivir-resistant viruses become dominant in the cell culture–propagated viruses. Chan et al. reported a similar case in which the original clinical specimens contained a mixed population of variants, and oseltamivir-resistant viruses become dominant after the passage in MDCK cells (*4*).

On his 9th day in the hospital, the patient was intubated because of acute respiratory distress syndrome (Figure) and given levofloxacin. Urine samples were negative for *Pneumococcus* and *Legionella* spp. antigens. The patient improved and was extubated on hospital day 16.

**Figure:**
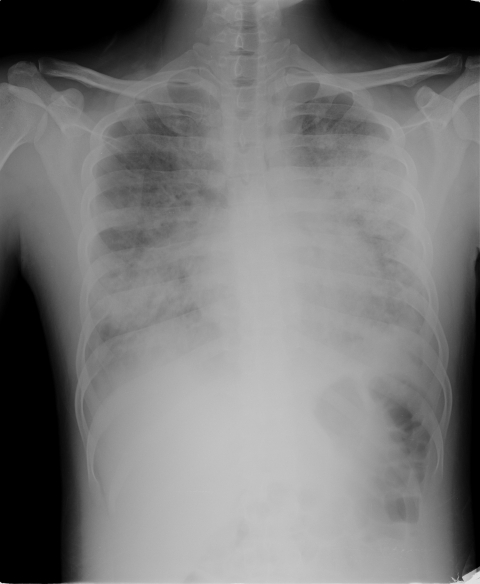
Radiograph (anteroposterior view) of patient with acute respiratory distress syndrome and oseltamivir-resistant pandemic (H1N1) 2009 virus.

Paired serologic test results were negative for *Mycoplasma pneumoniae* and *Legionella* spp. antibody; however, immunoglobulin G for *Chlamydia pneumoniae* increased 4-fold. By 37 days after illness onset, clinical signs and symptoms resolved and bilateral lineoreticular infiltration was reduced.

On August 8, 2009, Taiwan had the most devastating typhoon (Typhoon Morakot) in 50 years. The patient reported here had stayed in a typhoon evacuation camp for 1 week before his influenza signs and symptoms developed. Although 4 sporadic cases of pandemic (H1N1) 2009 infections were reported from the same camp, none of the isolated viruses harbored the H275Y mutation in the neuraminidase gene. No evidence of virus transmission was found among healthcare personnel, family members, and camp members who had been in close contact with the patient.

Oseltamivir has been recommended by the US Centers for Disease Control and Prevention for the treatment of infection caused by pandemic (H1N1) 2009 virus (*5*). The first 2 cases of oseltamivir resistance of pandemic H1N1 (2009) virus were reported in August 2009 (*6*). For these cases, oseltamivir-resistant virus was isolated on days 11 and 23 after the initial isolation of oseltamivir-susceptible viruses, for each patient, respectively. In contrast, in the case reported here, resistance to oseltamivir developed rapidly, after only 4 days of treatment.

In severe cases of pandemic (H1N1) 2009 infections, mortality rates are highest for patients who are pregnant, <2 years of age, or obese, or who have chronic lung disease (*7*). The patient reported here was previously healthy except for mental retardation; his body mass index was 23.9 kg/m^2^. Progression of pneumonia to acute respiratory distress syndrome occurred despite early initiation of the standard dose of oseltamivir, within 48 hours after illness onset and initial susceptibility of the virus. Clinical deterioration might have resulted from the rapid emergence of an oseltamivir-resistant pandemic (H1N1) 2009 virus with a H275Y mutation, which is known to confer a high level of oseltamivir resistance while retaining zanamivir susceptibility (*8*), or it might have resulted from co-infection with *C. pneumoniae*. A 4-month study found concurrent bacterial infections in 29% of fatal cases of pandemic (H1N1) 2009 virus (*9*).

Oseltamivir resistance can emerge rapidly during treatment of pandemic (H1N1) virus infection. Healthcare providers should be aware that resistance may emerge in otherwise apparently healthy persons as early as day 4 of treatment with standard doses of oseltamivir.
